# The Excellent Chemical Interaction Properties of Poloxamer and Pullulan with Alpha Mangostin on Amorphous Solid Dispersion System: Molecular Dynamics Simulation

**DOI:** 10.3390/polym16213065

**Published:** 2024-10-31

**Authors:** Agus Rusdin, Muchtaridi Muchtaridi, Sandra Megantara, Yoga Windhu Wardhana, Taufik Muhammad Fakih, Arif Budiman

**Affiliations:** 1 Department of Pharmaceutics and Pharmaceutical Technology, Faculty of Pharmacy, Universitas Padjadjaran, Jl. Raya Bandung-Sumedang Km. 21, Bandung 45363, Indonesia; agusrusdin@gmail.com (A.R.); y.w.wardhana@unpad.ac.id (Y.W.W.); 2 Department of Pharmaceutical Analysis and Medicinal Chemistry, Faculty of Pharmacy, Universitas Padjadjaran, Sumedang 45363, Indonesia; muchtaridi@unpad.ac.id (M.M.); s.megantara@unpad.ac.id (S.M.); 3 Department of Pharmacy, Faculty of Mathematics and Natural Sciences, Universitas Islam Bandung, Bandung 40116, Indonesia; taufikmuhammadf@gmail.com

**Keywords:** alpha mangostin, poloxamer, pullulan, amorphous solid dispersion, molecular dynamic

## Abstract

Background: Alpha mangostin (AM) has demonstrated significant potential as an anticancer agent, owing to its potent bioactivity. However, its clinical application is limited by poor solubility, which hampers its bioavailability and effectiveness. Amorphous solid dispersion (ASD) presents a promising technique to enhance the solubility and stability of AM. Molecular dynamics simulation offers a rapid, efficient, and precise method to evaluate and optimize ASD formulations before production. Aim of Study: In this study, we conducted molecular dynamics simulations to explore the ASD development of AM with poloxamer and pullulan. Result: Our results revealed that AM–poloxamer complexes exhibit superior interaction characteristics compared to AM–pullulan, with a 1:5 ratio of AM to poloxamer and a cooling rate of 1 °C/ns demonstrating the most favorable outcomes. This combination showed enhanced hydrogen bonding, a more compact molecular structure, and higher stability, making it the optimal choice for ASD formulation. Conclusion: The integration of molecular dynamics simulation into ASD development significantly accelerates the formulation process and provides critical insights into achieving a stable and effective AM dispersion. The AM–poloxamer complex, particularly at a 1:5 ratio with a 1 °C/ns cooling rate, offers the best potential for improving AM solubility and therapeutic efficacy.

## 1. Introduction

Alpha mangostin, a natural compound derived from the mangosteen fruit (Garcinia mangostana), has attracted considerable attention due to its strong pharmacological properties [1]. Its notable applications include demonstrated efficacy as an anticancer agent, particularly in breast cancer [2,3], as well as in other cancers such as prostate [4], cervical [5], and triple-negative breast cancer models [3,5,6,7]. Research indicates that alpha mangostin causes oxidative damage and mitochondrial dysfunction, resulting in apoptosis in cancer cells [7,8,9]. Additionally, it facilitates androgen receptor degradation in prostate cancer, presenting a new therapeutic strategy [4]. Additionally, the combination of this treatment with tamoxifen has been shown to improve antiproliferative effects on breast cancer cells and reduce endometrial side effects [10]. The compound downregulates critical oncogenes, including E6/E7, in HPV-related cervical cancer, thereby inhibiting tumor growth and proliferation [5]. Despite its promising attributes, the clinical application of alpha mangostin is constrained by poor solubility, which significantly limits its bioavailability and therapeutic potential [11]. The findings, supported by in vitro and in vivo studies, highlight alpha mangostin’s potential as a broad-spectrum anticancer agent, establishing a solid basis for further exploration of its solubilization strategies, including amorphous solid dispersion.

Amorphous solid dispersion (ASD) has emerged as a powerful strategy to address the solubility challenges of poorly water-soluble drugs like alpha mangostin [12,13]. By dispersing the active pharmaceutical ingredient (API) within an amorphous polymer matrix, ASD enhances both solubility and dissolution rates, thereby improving bioavailability [13]. The polymer carrier plays a crucial role in this process, as its properties—such as high glass transition temperature (Tg), miscibility with the API, and capacity to inhibit crystallization—are vital for stabilizing the amorphous state of the drug and maintaining its enhanced dissolution profile [14]. However, selecting the ideal polymer with the necessary attributes remains a key challenge in ASD development.

Molecular dynamics (MD) simulations provide an effective and systematic method to tackle the issues of generating amorphous solid dispersions (ASDs) by forecasting the molecular interactions between alpha mangostin and polymer carriers. This in silico approach offers a time-efficient and resource-conserving strategy for optimizing polymer selection and drug-to-polymer ratios, essential for preserving the amorphous state of poorly water-soluble pharmaceuticals. This method provides comprehensive insights into the physicochemical interactions of alpha mangostin with polymers like poloxamer and pullulan by simulating their molecular behavior, hence reducing the need for extensive experimental trials. This computational framework expedites the formulation creation process and establishes a foundation for targeted experimental validation, connecting molecular-level insights with practical ASD use [15,16].

In this investigation, alpha mangostin is incorporated with poloxamer and pullulan, owing to their demonstrated efficacy in prior research concerning amorphous solid dispersions (ASDs). Poloxamer, an amphiphilic copolymer [17], has exhibited remarkable solubilizing properties and stabilization of poorly soluble drugs in numerous studies, including its application in hot-melt extrusion ASDs for AMG 579, where it proficiently inhibited recrystallization and improved stability. Pullulan, a biocompatible and biodegradable polysaccharide [18,19], demonstrates considerable promise in the formation of stable amorphous systems and the enhancement of drug bioavailability, as illustrated by its application in drug delivery micelles for cancer therapy. In contrast to traditional polymers such as HPMC and PVP, poloxamer and pullulan exhibit enhanced solubility, stability, and biocompatibility, positioning them as exemplary choices for the stabilization of alpha mangostin in the development of amorphous solid dispersions.

The present study provides an innovative methodology employing molecular dynamics simulations for investigating amorphous solid dispersions (ASDs), an approach that is rarely encountered in the existing literature. Previous research have focused on clarifying the interactions between drugs and polymers through molecular dynamics simulation, represented by studies on Ritonavir–polymer systems [20], Rutin formulations [21], and alpha mangostin complexes [22,23]. For instance, studies such as the “Elucidation of Molecular Interactions Between Drug–Polymer in Amorphous Solid Dispersion” and the “Computational Approach to Elucidate the Formation and Stabilization Mechanism of Rutin Amorphous Formulation” used computational methods; however, there has been no investigation into the particular combination of alpha mangostin with poloxamer and pullulan polymers. Furthermore, despite research existing concerning the physical stability, dissolution, and self-assembly of alpha mangostin in solid dispersions, such as the study titled “Solid Dispersions of α-Mangostin Improve Its Aqueous Solubility through Self-Assembly of Nanomicelles,” there has been no in silico or experimental investigation into amorphous solid dispersion systems that utilize both poloxamer and pullulan polymers for alpha mangostin. This study addresses the existing gap by delivering an in-depth examination of molecular interactions through molecular dynamics simulations, thereby establishing a foundation for subsequent experimental endeavors for improving drug physicochemical properties and performance. This study aims to use molecular dynamics simulations to examine the chemical interaction properties of poloxamer and pullulan with alpha mangostin throughout an amorphous solid dispersion (ASD) system while discovering the optimal conditions to enhance the solubility and stability of alpha mangostin. This research enhances prior studies by utilizing in silico methodologies to offer a more efficient and accurate strategy for optimizing polymer selection and drug-to-polymer ratios, in contrast to conventional experimental methods. This study provides detailed molecular insights into the mechanisms of ASD formation, thereby expediting the development process and enhancing comprehension of how these systems can optimize the drug’s physicochemical properties, facilitating more targeted and informed experimental endeavors.

## 2. Materials and Methods

### 2.1. Alpha Mangostin and Poloxamer and Pullulan Structure Preparation

Prior to their utilization in MD procedures, the ligand structures were described and optimized. The molecular structures utilized in the present study were alpha mangostin, poloxamer, and pullulan, obtained from PubChem (http://pubchem.ncbi.nlm.nih.gov accessed on 21 November 2023). Following that, two-dimensional (2D) structures were delineated utilizing the ChemDraw Professional 16.0 software, while three-dimensional (3D) structures were acquired by transforming the 2D structures into 3D using the Chem3D 16.0 software. The ligand structure was optimized using GaussView 5.0.8 and Gaussian 09 software, employing the Density Functional Theory (DFT) approach with 6–31 basis sets. The polar hydrogen atoms and Gasteiger partial charge data were incorporated into the optimized structure using the AutoDock 4.2 algorithm with MGLTools 1.5.6.

### 2.2. Preparation of Melt Cooling Systems

The melt cooling method involved creating the system without the need for solvents. The study involved constructing a melt cooling system using a combination of alpha mangostin crystal molecules and poloxamer and pullulan molecules. The system was simulated in a box with dimensions of 5 Å × 4 Å × 4 Å, with a tolerance distance of 2 Å between the molecules. The system was developed using the PACKMOL 20.15.2. program, which is known for generating initial configurations for MD simulations through packing optimization.

### 2.3. Molecular Dynamics Simulation of Melt Cooling Systems

MD simulations were conducted on two complexes of melt cooling systems. The systems were distinguished by varying cooling rates: 1 °C/ns and 5 °C/ns. These rates were achieved using the Gromacs 2016.3 program, employing the AMBER99SB-ILDN and AMBER (GAFF) common force fields. The topology and molecular parameterization of AM and poloxamer and pullulan were generated using the Antechamber Python Parser interface (ACPYPE) program. Later on, the Ewald Particle Mesh method was employed to calculate long-distance electrostatic forces. The process of system neutralization involved the introduction of sodium (Na^+^) and chloride (Cl^−^) ions. During the preparation stage of MD simulations, various steps are undertaken, including minimization, temperature equilibrium, and pressure equilibrium. During the heating stage, Berendsen thermostats and barostats are utilized to maintain a pressure of 1 bar. This simulation utilizes a sizable canonical ensemble to accurately represent molecular systems. The large canonical ensemble is used to maintain a constant particle count (N), volume (V), and temperature (T) throughout the simulation. This allows for the investigation of molecular motion and thermodynamic properties. The process was furthered by conducting simulations (production run) using a 2 fs timestep. The production run process involved adjusting the temperature from 0 °C to 190 °C, with a heating rate of 30 °C/ns, and then maintaining it at 190 °C. The system was cooled to a temperature of 0 °C with different cooling rates of 1 °C/ns and 5 °C/ns (Table 1). Simulations were conducted for a duration of 500 ns on all systems until they achieved a stable state, as determined by the analysis of energy, pressure, temperature, and root mean square deviation (RMSD).

### 2.4. Analysis of Molecular Dynamics Simulation

Various calculations and analyses were performed on the complexes, including the root mean square deviation (RMSD), root mean square fluctuation (RMSF), radial distribution function (RDF), radius of gyration (Rg), solvent-accessible surface area (SASA), and number of hydrogen bonds. The calculations were conducted using Gromacs 2016.3 modules [24].

#### 2.4.1. Root Mean of Standard Deviation

The root mean square deviation (RMSD) is calculated using the formula below, which takes into account the number of atoms (N), the mass of each atom (mi), the start time of the simulation (t0), the current time (t), and the position of each atom (ri). The calculation was conducted following the completion of fitting using the least-squares technique.

$$\mathrm{RMSD}=\sqrt{\frac{\sum_{i=1}^{N}\mathrm{mif}(\mathrm{ri}\left(t\right)-\mathrm{ri}\left(t0\right)g^{2}}{\sum_{i=1}^{N}\mathrm{mi}}}$$

#### 2.4.2. Root Mean of Standard Fluctuation

RMSF is determined by the time interval between T0 and T, as well as the positions of the atoms at different time points. Specifically, it is calculated using the average position of atom i over time, denoted as [ri] t. The relationship between the root mean square fluctuation (RMSF) and B-factor Bi is defined as the RMSF of atom i, denoted as RMSFi.

$$\mathrm{RMSFi}=\sqrt{\frac{1}{T-T0}}\sum_{\mathrm{tj}=T0}^{T}{\left(\mathrm{ri}\left(\mathrm{tj}\right)-\left[\mathrm{ri}\right]t\right)}^{2}$$

$$\mathrm{RMSF}=\frac{8\pi^{2}}{3}{\mathrm{RMSFi}}^{2}$$

#### 2.4.3. Radial Distribution Function

The definition of RDF involves the distance between particles, the average number of atom pairs at specific distances, the total volume of the system, and the number of unique atom pairs.

$$g(r)=\underset{\mathrm{dr}\to 0}{\lim}\frac{p\left(r\right)}{4\pi \left(\frac{\mathrm{Npairs}}{V}\right)r^{2}\mathrm{dr}}$$

#### 2.4.4. Radius of Gyration

Rg is determined by the mass of atom i, mi, and its corresponding position, ri.

$$\mathrm{Rg}=\sqrt{\frac{\sum i=1m1r1^{2}}{\sum i=1m1}}$$

#### 2.4.5. Solvent-Accessible Surface Area

SASA is characterized by the surface area of each atom in an alpha mangostin and poloxamer and pullulan configuration and is determined by subtracting the patches shielded by close neighbor atoms from its maximum surface area. Within the realm of polymers, SASAi is a term used to describe the solvent-accessible surface area of an atom. This area represents the surface area of an individual sphere that is centered on the atom in question.

$$\mathrm{SASAi}=\mathrm{Constant}-{\mathrm{shielded}}_{j1}-{\mathrm{shielded}}_{j2}$$

## 3. Results

The study represents an in-depth molecular dynamics simulation of amorphous solid dispersions (ASD) of alpha-mangostin (AM) with poloxamer and pullulan polymers. The results highlight various structural, energetic, and conformational parameters, providing insight into the AM–polymer interactions.

### 3.1. Root Mean of Standard Deviation

**Figure 1:**
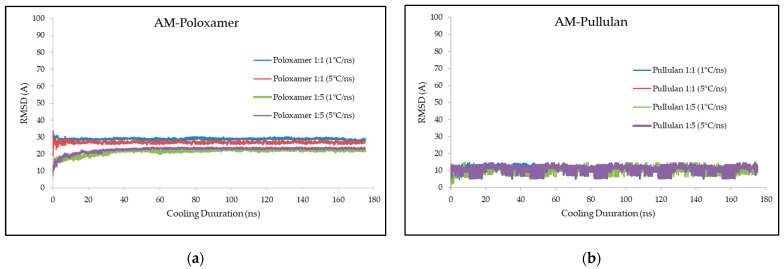
Illustrates the value of RMSD during molecular docking simulation between AM–poloxamer (**a**) and AM–pullulan (**b**). The x-axis represents cooling duration in nanoseconds, while the y-axis indicates the RMSD in Armstrong.

The RMSD analysis showed that cooling rate did not significantly influence the RMSD values in AM–poloxamer complexes, with values at 29 Å and 27 Å for 1 °C/ns and 5 °C/ns cooling rates, respectively, in a 1:1 ratio. In contrast, a 1:5 polymer-to-AM ratio reduced RMSD to 20 Å and 22 Å, emphasizing the polymer ratio’s role in complex stability. For AM–pullulan complexes, RMSD consistently measured at 10 Å across all conditions, suggesting minimal impact of polymer ratio and cooling rate on this system. Detailed values for each condition are shown in Figure 1.

### 3.2. Root Mean of Standard Fluctuation

**Figure 2:**
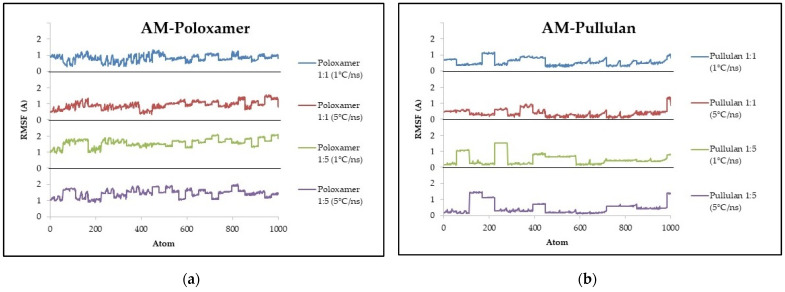
Illustrates the value of RMSF during molecular docking simulation between AM–poloxamer (**a**) and AM–pullulan (**b**). The x-axis represents atom/residue, while the y-axis indicates the RMSF in Armstrong.

The RMSF values for AM–poloxamer complexes ranged from 0.6 Å to 1.6 Å, with the lowest fluctuation observed at a 1:1 ratio and 1 °C/ns cooling rate (0.6 Å) and the highest at a 1:5 ratio and the same cooling rate (1.6 Å). The AM–pullulan complexes showed lower and consistent RMSF values between 0.3 Å and 0.5 Å across all conditions. Detailed fluctuations are shown in Figure 2.

### 3.3. Hydrogen Bonding Interaction

**Figure 3:**
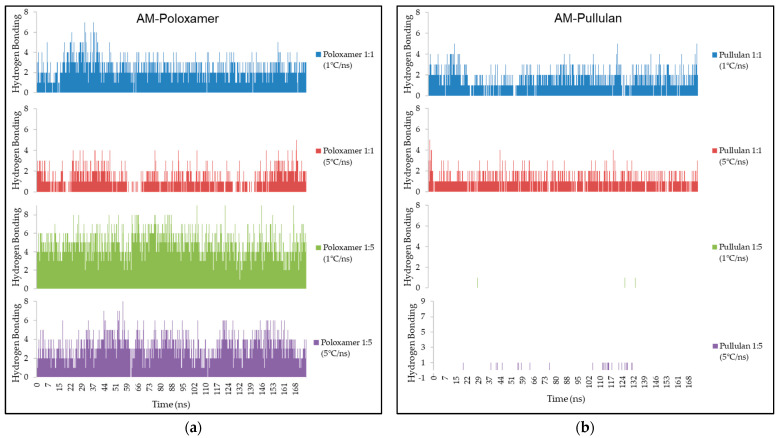
Illustrates the quantity of hydrogen bonding interactions established between AM–poloxamer (**a**) and AM–pullulan (**b**). The x-axis represents time in nanoseconds, while the y-axis indicates the amount of H-Bond.

In AM–poloxamer systems, the number and stability of hydrogen bonds varied with polymer ratio and cooling rate. A 1:1 ratio yielded four bonds (sustainability score of eight), while a 1:5 ratio at 1 °C/ns showed six bonds (sustainability score of ten), slightly reduced at 5 °C/ns to five bonds (score of seven). In AM–pullulan systems, hydrogen bonding characteristics remained stable under different conditions. Data illustrating these bonding interactions can be found in Figure 3.

### 3.4. Solvent-Accessible Surface Area

**Figure 4:**
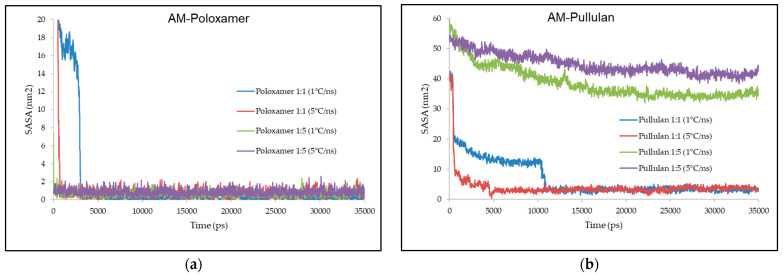
Illustrates the value of SASA during molecular docking simulation between AM–poloxamer (**a**) and AM–pullulan (**b**). The x-axis represents time in picoseconds, while the y-axis indicates the SASA in nm^2^.

AM–poloxamer complexes displayed SASA values from 0.6 to 0.9 nm^2^, increasing with higher polymer ratios and faster cooling rates. For AM–pullulan complexes, SASA values were significantly higher, ranging from 3 nm^2^ at a 1:1 ratio and 1 °C/ns to 42 nm^2^ at a 1:5 ratio and 5 °C/ns, indicating greater solvent exposure. These results are detailed in Figure 4.

### 3.5. Energy Interaction

Energy interactions varied between systems, with the AM–poloxamer complex showing more negative interaction energy, signifying stronger binding than the AM–pullulan system. The increase in polymer ratio and cooling rate showed a slight elevation in energy interaction values for both systems, but AM–poloxamer maintained lower energy values overall. A full summary of total energy interactions is provided in Table 2.

### 3.6. Radial Distribution Function

**Figure 5:**
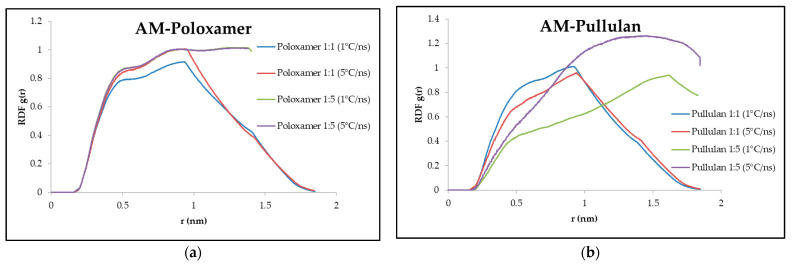
Illustrates the value of RDF value during molecular docking simulation between AM–poloxamer (**a**) and AM–pullulan (**b**). The x-axis (horizontal) represents the distance between atoms/molecules (in nanometers), and the y-axis (vertical) represents the probability of finding them at those distances.

In AM–poloxamer complexes, peak heights were consistent across cooling rates with a slight increase at higher cooling rates (0.85 at 1 °C/ns vs. 1 at 5 °C/ns for a 1:1 ratio). The AM–poloxamer 1:5 ratio displayed a broader peak width (2.7 nm) centered at 1.38 nm. For AM–pullulan complexes, a 1:5 ratio at 5 °C/ns displayed a peak height of 1.2, with broader widths indicating dispersed interactions. RDF results are presented in Figure 5.

### 3.7. Gyration Radius

**Figure 6:**
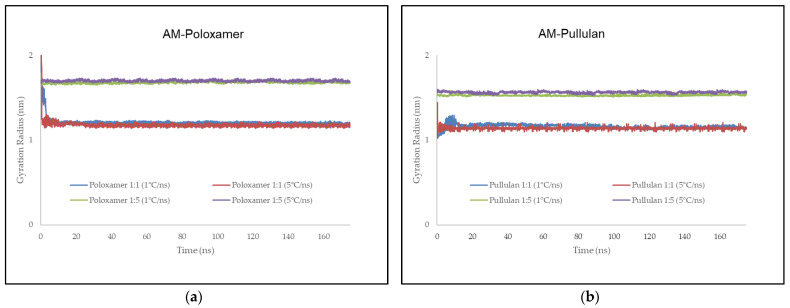
Illustrates the value of the gyration radius during molecular docking simulation between AM–poloxamer (**a**) and AM–pullulan (**b**). The x-axis represents time in nanoseconds, while the y-axis indicates the gyration radius in nm.

For AM–poloxamer complexes, the GR increased with polymer ratio and cooling rate, with values of 1.22 nm at 1 °C/ns and 1.20 nm at 5 °C/ns for the 1:1 ratio, and 1.72 nm and 1.75 nm for the 1:5 ratio. Similar trends were observed in AM–pullulan complexes, with GR values of 1.20 nm and 1.19 nm for the 1:1 ratio and higher values of 1.60 nm and 1.62 nm for the 1:5 ratio, suggesting an expanded conformation at higher ratios and cooling rates. These measurements are displayed in Figure 6.

## 4. Discussion

### 4.1. Root Mean Standard Deviation (RMSD)

This study employed the root mean square deviation (RMSD) to evaluate the structural stability and conformational dynamics of alpha mangostin (AM) complexes with poloxamer and pullulan polymers through molecular dynamics simulations (Figure 1). The RMSD values provide a quantification of the extent to which the molecular structures deviate from their original configurations [21,25]. Higher RMSD values indicate more significant structural fluctuations and potential instability, whereas lower values suggest stronger interactions and stability within the complex [26]. The results showed that the cooling rate had no significant effect on the RMSD for the AM–poloxamer complexes. At cooling rates of 1 °C/ns and 5 °C/ns, the observed RMSD values were 29 Å and 27 Å, respectively, for a 1:1 polymer-to-AM ratio. Nevertheless, a significant reduction in RMSD was observed as the polymer-to-AM ratio increased to 1:5, leading to RMSD values of 20 Å and 22 Å for the identical cooling rates. The polymer ratio is a critical factor in stabilizing the AM–poloxamer complex. On the other hand, the AM–pullulan complexes consistently showed RMSD values of 10 Å under all tested conditions. This indicates that the structural stability of the AM–pullulan system is not significantly affected by either the polymer-to-AM ratio or the cooling rate. When comparing the two polymers, it is clear that pullulan offers stronger stabilization of AM. This is demonstrated by its consistently low RMSD values, regardless of the experimental conditions. The observed difference can be explained by the inherent structural properties of pullulan [27]. Due to its multiple hydroxyl groups [19], pullulan forms extensive hydrogen bonds with AM, resulting in more stable interactions. However, poloxamer, a block copolymer [17], demonstrates a certain level of flexibility which leads to increased RMSD values, especially when the polymer ratios are lower. The findings partially support the initial hypothesis, indicating that both polymer type and formulation parameters have a significant impact on RMSD. Nevertheless, the cooling rate did not have the expected effect, especially in the poloxamer system, as the RMSD showed little variation. Higher RMSD values in the poloxamer system at lower ratios may be attributed to a lack of sufficient polymer to fully stabilize AM [28]. On the other hand, the pullulan system exhibits strong hydrogen bonding interactions, resulting in consistently low RMSD values and demonstrating effective stabilization. The observations underscore the significance of carefully selecting and optimizing the ratio of polymers in formulating amorphous solid dispersions. This is especially crucial in improving the stability and bioavailability of active pharmaceutical ingredients such as alpha mangostin.

### 4.2. Root Mean Square Fluctuation

The RMSF is an important parameter in molecular dynamics simulations, offering valuable information about the flexibility and dynamic behavior of atoms or residues within a molecular complex over time [29]. The RMSF calculation provides a comprehensive understanding of the structural dynamics at equilibrium by quantifying the average deviation of each atom or residue from its mean position during the simulation [30]. Regions of the molecule with lower RMSF values tend to be more rigid and stable, whereas higher values indicate increased flexibility or structural fluctuations [26,30]. The study found that there were noticeable differences in the behaviors of the AM–poloxamer and AM–pullulan complexes (Figure 2). These differences were influenced by factors such as the ratio of polymer to AM and the rate at which the samples were cooled. The RMSF values of the AM–poloxamer complexes varied between 0.6 Å and 1.6 Å. Among the different ratios tested, the 1:1 ratio at a cooling rate of 1 °C/ns exhibited the lowest fluctuation (0.6 Å), while the 1:5 ratio at the same cooling rate showed the highest fluctuation (1.6 Å). The results suggest that higher polymer ratios lead to increased flexibility of the complex, while the cooling rate does not consistently affect it. In contrast, the AM–pullulan complexes displayed significantly lower RMSF values, consistently ranging between 0.3 Å and 0.5 Å across all conditions. This suggests that these complexes have reduced structural fluctuations and greater overall stability when compared to the AM–poloxamer complexes.

When analyzing the importance of these variations, it is noteworthy to observe the differences in RMSF within the AM–poloxamer system. The range of 0.6 Å to 1.6 Å indicates that changes in the polymer ratio can have a significant impact on the flexibility of the complex [31]. Nevertheless, the absence of a distinct pattern linked to the cooling rate, and the limited variability observed in the AM–pullulan system, indicates that these factors may not have as significant an impact as originally hypothesized. The comparison between the two polymers highlights the superior stability of the AM–pullulan complexes. These complexes consistently exhibit lower RMSF values, indicating a strong interaction network within pullulan that restricts atomic fluctuations. It was hypothesized that the type of polymer, the ratio of polymer to AM, and the cooling rate would have a significant impact on the RMSF values, indicating a correlation with the stability of the complex. Although the findings in the poloxamer system provide some support, it is worth noting that the expected impact of cooling rate is not observed in the pullulan system. This suggests that flexibility in the poloxamer system is modulated by the polymer ratio, but further investigation is needed to understand the lack of impact on the cooling rate in the pullulan system. The low RMSF values observed in the AM–pullulan complexes can be attributed to the extensive hydrogen bonding of pullulan and its capacity to maintain a stable conformation [32,33,34]. This leads to reduced atomic fluctuations, regardless of the cooling rate or polymer ratio. In contrast, the properties of poloxamer are influenced by the ratio of polymer chains and their interaction with AM. As a result, higher concentrations of the polymer lead to increased RMSF values.

In summary, the results of the RMSF analysis indicate that pullulan exhibits a stronger and more consistent interaction with AM, leading to reduced atomic fluctuations and, consequently, a potentially more stable amorphous solid dispersion. The observed variations in the poloxamer system, especially when the polymer ratio is increased, indicate that the ability of poloxamer to stabilize AM is not consistently reliable and may lead to increased flexibility within the complex. The findings are consistent with the RMSD results, which showed that higher RMSD values in the poloxamer system at lower ratios indicated structural instability. This aligns with the increased RMSF values, which reflect greater atomic fluctuations. On the other hand, the consistently low RMSD and RMSF values observed in the Pullulan system provide further evidence of Pullulan’s exceptional stabilization properties. The relationship between RMSF and RMSD values underscores the link between molecular flexibility and overall structural stability, underscoring the significance of these parameters in the design of robust and efficient drug delivery systems.

### 4.3. Hydrogen Bonding Interaction

Expanding upon the earlier discussions of RMSD and RMSF, which offered valuable insights into the structural stability and flexibility of the AM–poloxamer and AM–pullulan complexes, the analysis of hydrogen bonding interactions provides additional clarity on the molecular interactions that contribute to these properties (Figure 3). Hydrogen bonding plays a crucial role in stabilizing molecular complexes, especially in amorphous solid dispersions. Strong and long-lasting hydrogen bonds have the potential to greatly improve stability and decrease molecular mobility. Greater values in the average amount of hydrogen bonding indicate stronger interactions, while the sustainability score reflects the persistence of these bonds over time during the simulation, with higher scores indicating more durable interactions [35,36].

The findings indicate that in the AM–poloxamer system, the quantity and durability of hydrogen bonds were influenced by both the polymer ratio and cooling rate. When the polymer-to-AM ratio was 1:1, the hydrogen bonding formed an average of four bonds with a sustainability score of eight out of ten. However, when the cooling rate increased to 5 °C/ns, the number of bonds decreased to three with a lower sustainability of five out of ten. On the other hand, when the polymer ratio was increased to 1:5, there was a notable improvement in hydrogen bonding. This resulted in the formation of six bonds and a sustainability score of ten out of ten when the cooling rate was slowed down to 1 °C/ns. Nevertheless, the interaction experienced a slight decrease when the cooling rate was raised to 5 °C/ns, leading to the formation of five bonds and a sustainability score of seven out of ten. Based on these observations, it can be inferred that a greater concentration of polymer in poloxamer, especially when cooled at a slower rate, promotes stronger and more stable hydrogen bonding interactions with AM.

In the AM–pullulan system, the strength and sustainability of hydrogen bonding were generally lower when compared to poloxamer. With a 1:1 ratio and a cooling rate of 1 °C/ns, the hydrogen bonding exhibited an average of three bonds and a sustainability score of six out of ten. However, these numbers decreased to two bonds and a sustainability score of five and a half out of ten when the cooling rate was increased. It is worth noting that when the polymer ratio was increased to 1:5, there was a significant decrease in hydrogen bonding. The sustainability scores also decreased to one out of ten and two out of ten at the slower and faster cooling rates, respectively. The results suggest that pullulan does not form or maintain hydrogen bonds with AM as effectively as poloxamer, particularly at higher concentrations and faster cooling rates.

The results indicate that poloxamer demonstrates superior efficacy compared to pullulan in establishing and preserving hydrogen bonds with AM. This aligns with previous observations of lower RMSD and RMSF values for poloxamer at higher ratios, indicating enhanced structural stability and decreased flexibility. The hypothesis predicted that the type of polymer, the ratio of polymer to AM, and the rate of cooling would have a significant impact on hydrogen bonding [37]. This is confirmed by the observed results in the poloxamer system. Lower hydrogen bonding in the pullulan system, especially at higher ratios and faster cooling rates, could be attributed to steric hindrance or a limited number of hydrogen bond donors and acceptors [38,39] within pullulan when interacting with AM.

The findings of this study indicate that poloxamer exhibits greater efficacy in forming and maintaining hydrogen bonds with alpha mangostin (AM), a conclusion that is further corroborated by prior research on analogous systems. A molecular dynamics (MD) simulation study on indomethacin–poly(vinylpyrrolidone) (PVP) systems demonstrated that cooling rates significantly influenced molecular mobility and hydrogen bonding dynamics, especially during the formation of amorphous solid dispersions (ASDs). In this context, accelerated cooling rates diminished hydrogen bonding interactions and heightened molecular disorder. This observation corresponds with the finding that poloxamer exhibits stronger hydrogen bonding interactions, particularly in comparison to pullulan at elevated ratios and increased cooling rates [40]. This phenomenon resembles the behavior of amorphous indomethacin–PVP systems, in which rapid cooling resulted in decreased stability and diminished hydrogen bonding interactions [41]. The influence of polymer ratios on hydrogen bonding interactions is extensively documented in numerous computational studies. The investigation of ritonavir–poloxamer ASDs via MD simulations indicated that an increase in polymer content resulted in enhanced hydrogen bonding interactions, thereby improving stability and molecular interactions within the system [20]. The observed pattern aligns with the study’s findings, indicating that increased polymer ratios of poloxamer improved hydrogen bonding with AM, thereby enhancing the stability and solubility of the ASD [20]. The complementary findings underscore the significance of cooling rates and polymer ratios in affecting the molecular stability of ASDs [42]. The quantity and long-term viability of hydrogen bonds play a crucial role in determining the overall stability and flexibility of the complex, as previously mentioned. The poloxamer system’s higher number and sustainability of hydrogen bonds are associated with its ability to maintain lower RMSD and RMSF values. This contributes to a more stable and less flexible complex. On the other hand, pullulan exhibits a lower hydrogen bonding capacity, especially at higher ratios. This leads to increased flexibility and reduced stability, which is evident from the higher RMSF values and the consistently low but stable RMSD values. The correlations mentioned emphasize the significance of hydrogen bonding in influencing the molecular dynamics and overall stability of amorphous solid dispersions. Poloxamer, in particular, exhibits strong stabilization properties due to its robust hydrogen bonding interactions.

### 4.4. Solvent-Accessible Surface Area (SASA)

Continuing from the earlier discussions on RMSD, RMSF, and hydrogen bonding interactions, which shed light on the stability, flexibility, and molecular interactions within the AM–poloxamer and AM–pullulan complexes, the assessment of the solvent-accessible surface area (SASA) provides additional insights into the interaction of these complexes with their surroundings (Figure 4). The SASA value of a molecule reflects the amount of surface area that is available to a solvent. Higher SASA values suggest greater exposure of the molecule to the solvent, which is often linked to less compact and more extended molecular conformations [43,44].

The results demonstrate a noticeable differentiation in the SASA values of the AM–poloxamer and AM–pullulan systems. The AM–poloxamer complex exhibited SASA values ranging from 0.6 to 0.9 nm^2^, indicating a progressive rise with increased polymer ratios and accelerated cooling rates. At a cooling rate of 1 °C/ns, the SASA was 0.6 nm^2^, which increased to 0.7 nm^2^ when the cooling rate was increased to 5 °C/ns. The trend was more noticeable when the ratio was 1:5. At a cooling rate of 1 °C/ns, the SASA increased from 0.8 nm^2^ to 0.9 nm^2^ at 5 °C/ns. The SASA values indicate that the AM–poloxamer complex has a compact conformation and limited exposure to the solvent. This aligns with the earlier findings of lower RMSD and RMSF values, as well as stronger hydrogen bonding interactions in this system.

On the other hand, the AM–pullulan complex showed considerably greater SASA values, suggesting a more elongated and exposed conformation in the presence of a solvent. With a 1:1 ratio and a cooling rate of 1 °C/ns, the surface area to volume ratio (SASA) measured 3 nm^2^. However, when the cooling rate was increased to 5 °C/ns, the SASA increased to 4 nm^2^. At a 1:5 ratio, the SASA values experienced a significant surge, reaching 36 nm^2^ and 42 nm^2^ at cooling rates of 1 °C/ns and 5 °C/ns, respectively. The observed increase in SASA with higher polymer ratios and faster cooling rates indicates that the AM–pullulan complex becomes less compact and more exposed to the solvent. This is consistent with the lower hydrogen bonding and higher RMSF values previously observed, suggesting greater molecular flexibility and reduced stability [26,45].

The results indicate that the choice of polymer, as well as the ratio of polymer to AM and the rate of cooling, have a substantial impact on the surface area of the complexes [46,47]. The hypothesis regarding the impact of polymer ratio and cooling rate on SASA has been confirmed in both systems. However, the effect is more significant in the AM–pullulan complex. The increased SASA observed in the pullulan system may be attributed to the naturally less compact structure of pullulan when it interacts with AM. This leads to a more elongated conformation and increased exposure to the solvent [46,47]. However, the comparatively low SASA observed in the poloxamer system is consistent with its capacity to establish stronger and longer-lasting hydrogen bonds with AM, resulting in a more condensed and stable structure [46,47].

The relationship between SASA and the properties mentioned earlier, including RMSD, RMSF, and hydrogen bonding, underscores the significance of molecular interactions in influencing the overall stability and flexibility of these complexes [24,48]. The SASA in the poloxamer system is lower, indicating a more stable and less flexible complex. This is supported by its lower RMSD and RMSF values and higher hydrogen bonding. On the other hand, the pullulan system has a higher SASA, suggesting greater flexibility, less stability, and weaker hydrogen bonding. The results underscore the significance of carefully choosing the right polymer type and processing conditions to enhance the stability and performance of amorphous solid dispersions in pharmaceutical applications.

### 4.5. Total Energy Interaction

Expanding upon the earlier examinations of RMSD, RMSF, hydrogen bonding interactions, and SASA, the examination of total energy interaction offers a thorough understanding of the thermodynamic stability of the AM–poloxamer and AM–pullulan complexes. The total energy, which encompasses the polar solvation energy, SASA energy, electrostatic energy, and van der Waals energy (Table 2), plays a vital role in assessing the overall interaction strength between the active molecule and the polymer matrix in molecular dynamic simulations [49,50]. Within this context, total energy values that are lower in magnitude indicate interactions that are more stable and favorable, whereas higher values suggest less stable interactions [49,51].

The findings demonstrate a clear distinction between the AM–poloxamer and AM–pullulan complexes in relation to overall energy levels. In the AM–poloxamer system, the total energy values consistently showed more negative values. The most stable configuration was found at a 1:5 ratio and a cooling rate of 1 °C/ns, resulting in a total energy of −972.9 kcal/mol. The stability of the system shows a slight decrease when the cooling rate is increased to 5 °C/ns, resulting in a less negative total energy of −826.9 kcal/mol. The total energy values at a 1:1 ratio were −411.8 kcal/mol at 1 °C/ns and −408.6 kcal/mol at 5 °C/ns. The results suggest that increasing the polymer ratio results in more stable interactions. Additionally, the cooling rate has an effect on stability, although its influence is not as strong as that of the polymer ratio.

On the other hand, the AM–pullulan system showed significantly lower total energy values, suggesting less stable interactions in general. The total energy at a 1:1 ratio was −317.8 kcal/mol at 1 °C/ns and −254.2 kcal/mol at 5 °C/ns. At a 1:5 ratio, the stability experienced a significant decrease. The total energy values were −37.3 kcal/mol at 1 °C/ns and −51.2 kcal/mol at 5 °C/ns. These findings indicate that in the AM–pullulan system, a higher polymer ratio results in a significant decrease in stability, which is in contrast to the observations made in the AM–poloxamer system. In addition, the cooling rate seems to have a more intricate impact, as there is a slight enhancement in stability when the higher cooling rate is applied in the 1:5 ratio configuration.

The differences between the two polymer systems can be explained by the unique properties of poloxamer and pullulan and their interaction with AM. Poloxamer, with its stronger hydrogen bonds and more compact structure (as seen in its lower SASA values), can establish more stable interactions with AM, especially at higher ratios. This supports the idea that a polymer matrix that is more compact and well-structured would lead to lower total energy values, indicating it is stronger and more favorable [50]. However, the AM–pullulan complex demonstrates higher surface area values and weaker hydrogen bonding, leading to less stable interactions, particularly at higher polymer ratios. The decrease in stability observed with a higher pullulan ratio could be attributed to pullulan’s limited ability to form a well-structured matrix that can efficiently interact with AM, resulting in a less favorable thermodynamic profile [52,53].

The relationship between the total energy values and the parameters mentioned earlier provides additional evidence that the stability of the AM–polymer complexes is closely tied to their structural and interactional characteristics. The lower total energy in the poloxamer system is associated with its lower RMSD, RMSF, and SASA values, as well as its stronger hydrogen bonding. These findings suggest a more stable and less flexible complex. In contrast, the pullulan system exhibits a higher total energy, which corresponds to its higher RMSD, RMSF, and SASA values. Additionally, the weaker hydrogen bonding in this system indicates a less stable and more flexible complex. The findings highlight the significance of carefully choosing the polymer type and processing conditions to enhance the stability and efficacy of amorphous solid dispersions in pharmaceutical applications.

### 4.6. Radial Distribution Function (RDF)

The Radial Distribution Function (RDF) offers valuable insights into the molecular structure and interactions within the system. It demonstrates the variation in particle density as distance from a reference particle changes [54]. Within this context, a high RDF peak signifies a greater likelihood of discovering nearby molecules at a particular distance, indicating a robust local structuring or interactions [55]. Conversely, a broader peak implies a more scattered and less organized arrangement. Sharp and elevated peaks generally indicate clear and robust interactions between the molecules, while broader and lower peaks suggest weaker or more dispersed interactions [56].

The results of the RDF analysis indicate that the peak heights of the AM–poloxamer complexes remain consistent at both cooling rates. However, a slightly higher peak is observed at the higher cooling rate (1 at 5 °C/ns compared to 0.85 at 1 °C/ns for the 1:1 ratio). The peak width is broader at the higher AM–poloxamer ratio (1:5), suggesting a wider range of interaction (Figure 5). This is particularly noticeable with the broader width (2.7 nm) at both cooling rates, which corresponds to a shift in the center peak to 1.38 nm. On the other hand, the AM–pullulan complexes exhibit a distinct pattern. The RDF peak height shows a slight decrease as the cooling rate increases for the 1:1 ratio, but a significant increase for the 1:5 ratio (reaching up to 1.2 at 5 °C/ns). The peaks observed for the 1:5 ratio in AM–pullulan complexes indicate a more dispersed interaction profile. This is evident in the wider peak at 3.7 nm, centered at 1.3 nm.

The RDF results of the two polymers reveal that AM–poloxamer exhibits more consistent interaction distances. Its peak ranges are narrower and peak heights remain relatively stable, suggesting stronger and more localized interactions [54]. In contrast, AM–pullulan shows wider peaks, particularly at higher AM–polymer ratios, suggesting more dispersed and less concentrated interactions [57]. This aligns with the previous discussions on total interaction energy and hydrogen bonding. AM–poloxamer exhibited stronger interactions, as evidenced by its more negative total energy and greater hydrogen bonding sustainability. The broader RDF peaks observed in AM–pullulan are consistent with its comparatively less negative total energy, indicating a weaker overall interaction [58].

The results support the hypothesis that higher ratios of AM to polymer and faster cooling rates could result in more extended and less defined interaction networks [59,60]. This is especially evident in the case of pullulan, where the interactions are more dispersed. The RDF is greatly affected by the polymer type, ratio, and cooling rate, as they play a crucial role in determining the molecular arrangement and interaction strength within the amorphous solid dispersion system [61,62]. The correlation between RDF, total energy, and hydrogen bonding provides additional evidence for the conclusion that poloxamer promotes stronger and more localized interactions compared to pullulan. This finding is in line with poloxamer’s role in improving the stability and efficacy of the amorphous solid dispersion.

### 4.7. Gyration Radius (GR)

The gyration radius (GR) provides insight into the overall compactness or expansion of a molecular system. It measures the average distance of the components of a system from their center of mass, with higher GR values indicating a more expanded or less compact structure, while lower GR values suggest a more compact or condensed structure [63,64].

In the results provided, the AM–poloxamer complexes show a trend where the GR increases with both a higher AM to polymer ratio and a higher cooling rate. Specifically, for the 1:1 ratio, the GR is relatively stable (1.22 nm at 1 °C/ns and 1.20 nm at 5 °C/ns), whereas, for the 1:5 ratio, it increases to 1.72 nm and 1.75 nm, respectively. This suggests that a higher polymer ratio and faster cooling rate lead to a more expanded conformation. Similarly, the AM–pullulan complexes exhibit a similar trend, with GR values of 1.20 nm and 1.19 nm for the 1:1 ratio and 1 °C/ns and 5 °C/ns cooling rates, respectively, and higher GR values of 1.60 nm and 1.62 nm for the 1:5 ratio at the corresponding cooling rates. This indicates that the AM–pullulan systems also become more expanded with increasing AM ratio and cooling rate (Figure 6).

Comparing the two polymers, AM–poloxamer consistently shows slightly higher GR values compared to AM–pullulan at equivalent ratios and cooling rates, particularly noticeable at the 1:5 ratio. This suggests that AM–poloxamer tends to form a more expanded structure compared to AM–pullulan, which may relate to its interactions and binding with the polymer.

These results align with the hypothesis that higher AM to polymer ratios and faster cooling rates can lead to more expanded molecular conformations [65]. The increase in GR with the higher ratio suggests that more AM molecules disrupt the compactness of the polymer network, leading to a more extended structure [66]. Additionally, faster cooling rates may inhibit the ability of the system to reach a more compact, ordered state, leading to larger GR values [65,66].

In correlation with previous results, the GR values support the findings from the total energy, hydrogen bonding, and RDF analyses. For example, the higher GR observed for AM–poloxamer aligns with its stronger total interaction energy and higher hydrogen bonding sustainability, indicating a more pronounced interaction with a more extended molecular structure. Conversely, the lower GR for AM–pullulan reflects its less negative total energy and lower hydrogen bonding sustainability, suggesting a more compact and less extended structure [65,66].

Overall, the gyration radius provides a valuable perspective on the conformational changes in the system, complementing the insights gained from total energy, hydrogen bonding, and RDF analyses. It highlights how the polymer type, ratio, and cooling rate influence the molecular arrangement and interaction within the amorphous solid dispersion system.

## 5. Study Limitation Insights

This study takes into account the limitations of molecular dynamics (MD) simulations, which, despite providing valuable insights into molecular interactions, possess inherent constraints in the in silico approach, especially when compared with actual experimental conditions. This work investigated the molecular interactions inside the amorphous solid dispersion (ASD) of alpha mangostin with poloxamer and pullulan polymers utilizing melt cooling methods. The obtained information, encompassing hydrogen bonding interactions, energy parameters, RMSD, RMSF, and various thermodynamic and molecular properties, offers a comprehensive insight into molecular interactions; however, these findings merely approximate the intricate behavior of the system in a real physical context.

A limitation is the degree of complexity that can be represented in molecular dynamics simulations. Despite utilizing force fields such as AMBER99SB-ILDN and AMBER (GAFF) to simulate interactions, these force fields are based on classical physics, potentially overlooking quantum mechanical effects that may be substantial at the atomic scale. Molecular interactions, particularly those involving electron transfer or polarization effects, necessitate advanced quantum mechanical analyses. The classical force fields employed in this study presume static charges and predetermined atomic types, which may not consistently reflect the dynamic alterations present in molecular systems under actual experimental conditions, including solvation effects, molecular flexibility, or fluctuations in ionic strength.

A further constraint stems from the cooling rates and heating techniques employed in molecular dynamics simulations. This work employed cooling rates of 1 °C/ns and 5 °C/ns, which offered insights into their impact on the system’s structural properties; nevertheless, these speeds exceed those attainable under experimental conditions. The disparity between simulated and experimental cooling rates may affect the extent of molecular ordering, crystallization, or amorphization within the system. The swift cooling utilized in MD simulations may result in structures that deviate from those created under experimental conditions, where cooling transpires at a far slower rate. This may influence the stability, shape, and physicochemical characteristics of the final amorphous solid dispersion.

Moreover, molecular dynamics simulations are intrinsically constrained by the timescales they can investigate. This work used simulations lasting 500 ns, a considerable duration in computational terms, yet significantly shorter than the timescales associated with molecular rearrangements and diffusion in actual systems. The constraint in timescale precludes the observation of long-term stability, recrystallization processes, or phase transitions that may transpire over days, weeks, or months inside an experimental system. Such constraints can affect the analysis of molecular interactions and thermodynamic characteristics, such as hydrogen bonding configurations or the prolonged stability of the ASD system.

Furthermore, the molecular parameters included in this study, including RMSD, RMSF, RDF, and gyration radius, offer valuable metrics for system analysis; nevertheless, these are ultimately based on a model that may not comprehensively encompass all real-world variables. The Ewald Particle Mesh method used to compute long-range electrostatic forces operates under a periodic boundary condition, which may not accurately reflect real molecular environments, particularly in systems with intricate geometries or boundary effects.

This study provides substantial benefits for comprehending molecular interactions and refining polymer selection for ASD development; however, the in silico methodology fails to consider external influences such as impurities or the mechanical stress encountered in experiments. The formation of hydrogen bonds and other interaction characteristics shown in the simulation may be affected by external solvation conditions, which are not consistently represented in vacuum or non-aqueous environments. Moreover, the computational models employed to mimic molecular behavior in silico may neglect nuanced yet essential environmental elements, such as humidity, which can profoundly influence the performance and stability of the amorphous system.

The shift from molecular dynamics simulations to experimental validation will be essential in verifying the reliability of the results. The computational analysis offers significant insights into molecular mechanisms; yet, it cannot entirely encompass the intricacies of experimental methods, like system scaling, material handling, or actual dissolution patterns. The subsequent phase of this project will concentrate on experimental laboratory work to corroborate these findings and reconcile the disparity between theoretical predictions and practical applications. This experimental study will validate the molecular interactions, optimize the drug–polymer ratios, and evaluate the enhancements in solubility and bioavailability anticipated by the MD simulations.

In conclusion, while our study establishes a robust basis for comprehending the molecular interactions in the alpha mangostin ASD, it is subject to constraints associated with the computational approaches employed. This encompasses the dependence on classical force fields, the divergence between simulated and experimental cooling rates, and the incapacity to comprehensively represent long-term molecular dynamics or environmental influences. It is imperative to validate these computational conclusions by experimental research to confirm the correctness and relevance of the findings in practical pharmaceutical development.

## 6. Conclusions

In conclusion, the molecular dynamics simulations conducted in this study have provided comprehensive insights into the molecular interactions between alpha mangostin and the selected polymers, poloxamer and pullulan, which are critical for advancing the development of amorphous solid dispersions (ASDs). The results indicate that poloxamer, specifically at a 1:5 drug-to-polymer ratio, is the most advantageous candidate for ASD formulation owing to its enhanced molecular interaction properties, which comprise stronger hydrogen bonding, a more compact molecular arrangement, and improved overall stability. The simulations indicate that a cooling rate of 1 °C/ns is best for achieving a well-ordered and stable dispersion, crucial for maintaining drug stability and improving its physicochemical properties. These findings provide a robust basis for subsequent experimental projects, wherein this in silico methodology will be applied to laboratory experiments to enhance and validate the effectiveness of the ASD system for alpha mangostin. This work is a vital advancement in reducing the time needed for polymer optimization and drug formulation, potentially enhancing the bioavailability and therapeutic efficacy of alpha mangostin.

## Figures and Tables

**Table 1:** Alpha mangostin–polymer complex in amorphous solid dispersion system.

Complex	Drug–Polymer	Mass Ratio (AM–Polymer)	Cooling Rate
Complex 1	AM–poloxamer	1:1	1 °C/ns
Complex 2			5 °C/ns
Complex 3		1:5	1 °C/ns
Complex 4			5 °C/ns
Complex 5	AM–pullulan	1:1	1 °C/ns
Complex 6			5 °C/ns
Complex 7		1:5	5 °C/ns
Complex 8			1 °C/ns

**Table 2:** Illustrates the energy interactions established between AM–poloxamer and AM–pullulan.

Energy	AM–poloxamer				AM–pullulan			
	Ratio (1:1)		Ratio (1:5)		Ratio (1:1)		Ratio (1:5)	
	(1 °C/ns)	(5 °C/ns)	(1 °C/ns)	(5 °C/ns)	(1 °C/ns)	(5 °C/ns)	(1 °C/ns)	(5 °C/ns)
Polar solvation	225.5	249.9	571.8	482.9	242.4	182.6	26.2	19.2
SASA	−63.9	−69.2	−128.6	−115.2	−50.8	−36.0	−6.3	10.6
Electrostatic	−73.5	−78.0	−205.8	−161.5	−64.9	−59.6	−3.3	−2.3
Van der Waals	−411.8	−511.4	−1210.4	−1933.1	−444.6	−341.0	−53.8	−57.5
Total energy	−411.8	−408.6	−972.9	−826.9	−317.8	−254.2	−37.3	−51.2

## Data Availability

The original contributions presented in the study are included in the article, further inquiries can be directed to the corresponding author.
